# THE HIGH RESOLUTION ESOPHAGEAL MANOMETRY AND PREDICTORS PARAMETERS OF DYSPHAGIA IN POST-LAPAROSCOPIC HIATOPLASTY AND NISSEN FUNDOPLICATION - A SYSTEMATIC REVIEW

**DOI:** 10.1590/S0004-2803.24612024-112

**Published:** 2025-09-05

**Authors:** Ary Augusto de Castro MACEDO, Danielle Patriota SAMPAIO, Natalie Cavalcanti Mareco da SILVA, Luigi Carlo da Silva COSTA, Nelson Adami ANDREOLLO, Luiz Roberto LOPES

**Affiliations:** 1 State University of Campinas, Faculty of Medical Sciences, Department of Surgery, Digestive Diseases Surgical Unit - Campinas (SP), Brazil.; 2 Medical Collaborator of Digestive System Disease Diagnostic Center of State University of Campinas (GASTROCENTRO - UNICAMP). Brazil.; 3 Coordinator of the Surgical Skills Laboratory, Integrated Health Department of the State University of Pará (UEPA). Brazil.

**Keywords:** Fundoplication, manometry, dysphagia, gastroesophageal reflux disease, Fundoplicatura, manometria, disfagia, doença do refluxo gastroesofágico

## Abstract

**Background:**

Gastroesophageal reflux disease has a prevalence of 12% in the Brazilian population. Its treatment includes hygienic-dietary changes, use of medications and, in selected cases, surgery with laparos­copic hiatoplasty and Nissen total fundoplication. However, this last treatment modality presents risks of postoperative dysphagia. High Resolution Esophageal Manometry (HREM) has been considered the test of choice for identifying patients who are candidates for surgical treatment at an increased risk of developing dysphagia.

**Objective:**

The objective of this study is to carry out a systematic review to evaluate the clinical and manometric factors that predict post-hiatoplasty and Nissen fundoplication dysphagia using HREM.

**Methods and results:**

Having defined the search engine, we used the databases MEDLINE, PUBMED, EBSCOHOST, SCOPUS and EMBASE. 2147 articles were identified. After selection, 11 studies remained.

**Conclusion:**

We concluded that the data from the selected articles are heterogeneous, but there is agreement regarding a higher risk of dysphagia among female patients, patients with dysphagia present in the preoperative period and, about manometric parameters, for patients with dysphagia in the preoperative period, there is a higher incidence of dysphagia resolution for patients with DCI >1000 mmHg.s.cm. In patients with ineffective esophageal motility, it is recommended to perform the Rapid Multiple Swallowing Test to assess the contractile reserve of the eso­phageal body. If there is an increase in contractile strength with this test, it is considered safe to perform a total fundoplication because the incidence of late dysphagia is low in these cases.

## HIGHLIGHTS

• There is great concern about dysphagia after laparoscopic hiatoplasty and fundoplication, but still lack predictors factors of preoperative tests.

• We carried out a systematic review aimming of evaluating the High Resolution Esophageal Manometry (HREM) and the predictors of dysphagia after laparoscopic hiatoplasty and Nissen fundoplication. Patients with negative multiple rapid swallow test (MRST) in the preoperative HREM is a predictor of postoperative dysphagia. Among patients with preoperative dysphagia, there is a considerable chance of it being resolved after hiatoplasty and fundoplication if they have an DCI >1000 mmHg.s.cm on the preoperative HREM.

• HREM plays a key role in the preoperative assessment of patients with GERD, excluding differential diagnoses and helping select patients with risk of postoperative dysphagia.

## RESUMO

**Contexto:**

A Doença do refluxo gastroesofágico tem prevalência de 12% na população brasileira. Seu tratamento inclui mudança higieno-dietéticas, uso de medicamentos e, em casos selecionados, a cirurgia com hiatoplastia e fundoplicatura total videolaparoscópica à Nissen. No entanto, esta última modalidade de tratamento apresenta riscos de disfagia no pós-operatório. A manometria esofágica de alta resolução (MEAR) tem sido colocada como o exame de escolha para identificação de paciente candidatos ao tratamento cirúrgico com risco aumentado de desenvolver disfagia.

**Objetivo:**

O objetivo deste estudo é a realização de uma revisão sistemática para avaliar os fatores clínicos e manométricos preditores de disfagia pós-hiatoplastia e fundoplicatura mediante utilização da MEAR.

**Métodos e resultados:**

Definido o mecanismo de busca, utilizamos das bases MEDLINE, PUBMED, EBSCOHOST, SCOPUS e EMBASE. Fora identificados 2147 artigos e, após seleção, restaram 11 estudos.

**Conclusão:**

Concluímos que os dados advindos dos artigos selecionados são heterogêneos, porém há concordância quanto a mais risco de disfagia dentre pacientes femininas, pacientes com disfagia presente no pré-operatório e, quanto aos parâmetros manométricos, para os pacientes com disfagia no pré-operatório, tem-se maior incidência de resolução da disfagia para paciente com DCI >1000 mmHg.s.cm. Nos pacientes portadores de motilidade esofagiana ineficaz, recomenda-se a realização do teste de múltiplas deglutições rápidas para se avaliar a reserva contrátil do corpo esofágico. Caso haja aumento da força contrátil com o referido teste, é considerado segura a realização de fundoplicatura total já que nestes casos é baixa a incidência de disfagia tardia.

## INTRODUCTION

Reflux of part of gastric contents into the esophagus in a retrograde and controlled manner is considered normal^1^. However, when it occurs excessively and causes symptoms associated with evidence of pathological, histological or endoscopic changes or abnormal esophageal pH monitoring, gastroesophageal reflux disease (GERD) is defined^2^. The main symptoms of this disease are heartburn and regurgitation, and national studies show that they are prevalent in 12% of the Brazilian population^3^. In the United States, heartburn is the symptom that most motivates people to go to gastroenterology clinics and it is present in 30% of patients at least twice a week^4^. 

The treatment of GERD consists lifestyle and dietary modification such as changes in sleeping position and eating routine to avoid or minimize gastroesophageal reflux and the use of medications, commonly proton pump inhibitors (PPIs) and potassium competitive acid blockers (P-CABs). In specific cases, concomitant hiatus hernia (HH) or other complications of GERD such as Barrett’s esophagus, surgical treatment is recommended with hiatoplasty and fundoplication. However, like all surgery, this procedure is not harmless and, with the fundoplication and increased pressure in the esophagogastric junction (EGJ), dysphagia can occur postoperatively in up to 12% of cases, according to the LOTUS Trial^5^. In order to optimize post-operative results and minimize the risk of dysphagia, it is necessary to exclude other diseases affecting the EGJ, such as achalasia or Chagas disease, and to carry out a detailed study of the contractile force of the esophageal body before proposing this treatment modality. For this assessment, esophageal manometry has proved useful, but there is still no consensus in the literature as its value in defining the surgical strategy to be employed. With the advent of high resolution esophageal manometry (HREM), expectations regarding the selection of patients for surgical treatment of GERD have become even higher. However, there is still lack of systematic and concordant scientific evidence to guide this practice. In view of this background, the aim of this study was to carry out a systematic review to find out what the scientific evidence is regarding manometric factors that predict dysphagia after video-laparoscopic hiatoplasty and fundoplication, specifically in relation to HREM parameters.

## METHODS

Preserving methodological rigor of a systematic review and achieve accurate results, this study was prepared in accordance to the recommendations of the Preferred Reporting Items for Systematic Reviews and Meta-Analyses Protocol (PRISMA-P). Using the PICOT method, the following key question was defined for the search engine: What are the manometric predictors of dysphagia after laparoscopic hiatoplasty and Nissen fundoplication? Search and eligibility criteria were defined as described below. 

To develop the search strategy, we searched the controlled vocabularies: DESC, MESH and EMTREE. After locating the descriptors, a search strategy was devised for PUBMED, where it was possible to check whether the articles retrieved were relevant and answered the review’s guiding question. By analyzing the articles retrieved from this preliminary search, we were able to observe some variations in terms that were used in the final search strategy to make it more sensitive so as not to miss important articles for the review. Once the search strategy had been defined, it was adapted to the following databases and portals previously selected for their relevance and scope: MEDLINE, PUBMED, PUBMED PMC, EBSCOHOST, SCOPUS, WEB OF SCIENCE and EMBASE. We did not limit the papers by year of publication, but only used articles in English or Portuguese. The terms selected were: (Fundoplication or “stomach fundoplication” or “fundal plication” or “fundic wrap” or “fundic wrapping” or “fundo-plication” or fundoplicatio or fundoplication or ‘fundoplicative maneuver’ or ‘fundoplicative surgery’ or fundoplicature or ‘stomach fundus plication’ or ‘Antireflux surgery’ or ‘antireflux operation’ or ‘antireflux operation’) and (manometry or ‘high resolution manometry’ or ‘high-resolution manometry’ or ‘esophageal manometry’ or ‘manometric investigation’) and “Postoperative Complications”. After searching the selected sources, we used the EndNote Web reference manager to exclude duplicates and the Rayyan tool to analyze the titles and abstracts of the articles by the three collaborators involved in this review. The study analysis was paired and blinded following the pre-established inclusion and exclusion criteria, as follows:

### Inclusion criteria

- Any design studies with adult patients regardless of gender or comorbidities;
- Patients with confirmed GERD by pHmetry or impedance-pHmetry, who underwent laparoscopic hiatoplasty and total Nissen fundoplication;
- Studies which preoperatively analysed patients with HREM;

### Exclusion criteria

- Studies with patients operated by other tech niques than laparoscopic hiatoplasty and total Nissen fundoplication;
- Studies that did not follow up patients for more than 3 months after surgery;
- Studies with reoperation of patients with GERD;
- Studies that did not evaluate dysphagia as a postoperative complication;
- Studies with children and elderly.

After the first stage of selection, the full texts articles were accessed in order to identify which of it used HREM. At this stage, the Rayyan tool was used again for selection as described above. Once the second selection stage had been completed, the inclusion and exclusion criteria were tested again and the remaining articles were read in full by one of the collaborators. The relevant informations were then extracted, tabulated in Excel software and a comparison was made between the articles and a descriptive table of the results of this systematic review was drawn up.

## RESULTS

**FIGURE 1:**
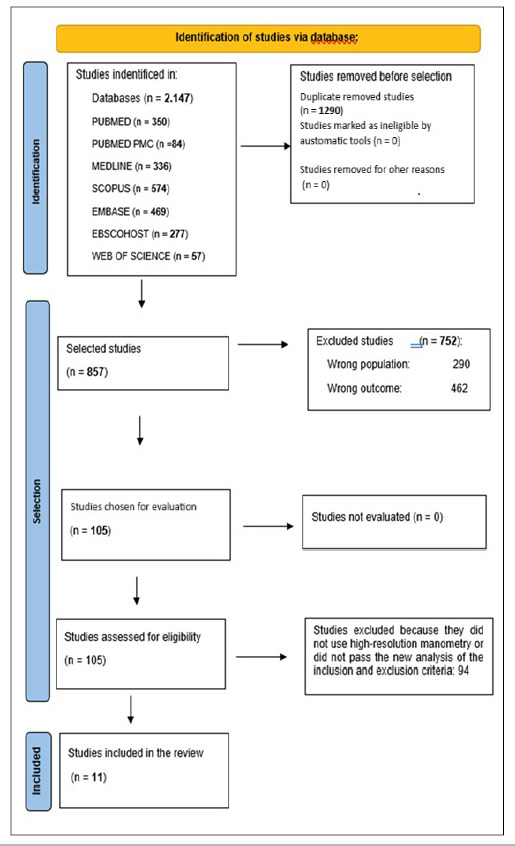
Flowchart identification, selection of studies in the literature and inclusion in the systematic review.

The search using the descriptors mentioned above identified 2147 articles. Excluding duplicates, 857 articles remained. In the first selection stage, 105 articles were included. After the second stage of selection by the 3 examiners, the remaining articles were again assessed in terms of inclusion and exclusion criteria. This left 11 articles that used HREM and met the inclusion and exclusion criteria, as shown in Figure 1. These 11 articles were fully read by one of the collaborators and the information extracted was tabulated and compared as shown in Table 1.

**TABLE 1:** Results of the systematic review.

Author	Year	Study design	N (with dysphagia)	Limitation	Predictors factors of dysphagia	Conclusions / additional informations
Winslow	2003	Analysis of prospective data	168 (22)	Analysis of HREM’s findings in disagreement with the currently standardization	A trend towards more frequent postoperative dysphagia in patients with esophageal motor disorders (28% with motor disorders vs. 14% in controls; p: 0.061)	Nonspecific esophageal motor disorder does not contraindicate antireflux surgery, but realistic expectations of symptom improvement should be set,
Scheffer	2005	Clinical trial	12 (11)	Use of a probe with only 16 channels	None	post-operative dysphagia does not correlate with direct manometric parameters, but rather with the transit time of the bolus through the EGJ
Broeders	2009	Randomized Clinical trial	148 (14)	Post-operative HREM only performed on a few patients in the study. Sample not representative of the whole population study	Among 14 patients with postoperative dysphagia, 8 of them had IEM preoperatively - without statistical significance;	Hiatoplasty with laparoscopic total fundoplication is the procedure of choice for GERD. Despite the higher incidence of early dysphagia, late dysphagia is the same as with conventional surgery
Candice	2012	Historical serie	14 (14)	Small sample size; absence of preoperative HREM in some patients; younger control patients; variations in the surgical techniques used	Preoperative dysphagia; female gender	Postoperative changes in the functional anatomy of the esophageal hiatus are the primary factor responsible for dysphagia after total fundoplication.
Shaker	2013	Prospective	63 (34 early; 18 late)	Small sample size; some patients did not finish the MRST; reproducibility of the MRST	Little increase in the force of contraction of the esophageal body in MRST is more prevalent in patients who develop late postoperative dysphagia	First study to propose MRST to assess contractile reserve and risk of dysphagia. MRST can be useful in terms of postoperative symptom expectation, especially in patients with borderline peristalsis.
Siegal	2018	Prostective review	94 (23)	The surgical technique used was defined according to the HREM; Esophageal contrasted X-ray was not used in this study	There were no HREM metrics predictive of dysphagia. In patients with preoperative dysphagia, a mean DCI > 1000mmHg.s.cm was noted in patients with resolved dysphagia.	Preoperative dysphagia is common and typically resolves after fundoplication, especially in those patients with DCI > 1000mmHg.s.cm.
Peixoto	2019	Prospective review	27 (27)	Small sample; patients with IEM or fragmented swallowing only underwent surgical treatment if they were MRST positive	Increased baseline LES pressure;	Our data reinforces the need for additional metrics to better identify patients at high risk of post-fundoplication dysphagia
Hasak	2019	Retrospective review	157 (101)	Data collection dependent on the patient’s attendance at follow-up; did not assess the use of PPIs in the postoperative period	Esophageal motility dysfunction with negative MRST predicts late post-fundoplication dysphagia as well as preoperative dysphagia and early dysphagia.	The assessment of preoperative dysphagia, in addition to the adequacy of the fundoplication, can be of additional value in preventing post-fundoplication dysphagia
Tageldin	2021	Prospective coorte	373 (90)	Total fundoplication in only 10.4% of patients; it does not specify the value of the DCI in question. Fundoplication chosen by the surgeon according to HREM	None	High postoperative DCI and IRP is related to postoperative dysphagia.
Tamises	2022	Retrospective review	206 (38)	Published text does not explain statistical calculations	There was no difference in the incidence of dysphagia in IEM patients according to the Chicago Classification 3.0 and 4.0 criteria	The narrower diagnosis of IEM by CC4.0 does not improve the ability to predict postoperative dysphagia
Salvador	2024	Historical serie	132	Small number of participants; surgical technique based on HREM; no endoscopy or esophageal contrasted X-ray performed with HREM in the postoperative period	No independent association between preoperative manometric measurements and fundoplication failure in multivariate analysis	Postoperative IRP correlated with the onset of dysphagia after the laparoscopic hiatoplasty and fundoplication

## DISCUSSION

The largest cohort of patients after laparoscopic hiatoplasty and fundoplication^6^, as two other studies^7^ ^,^ ^8^, point to the superiority of this technique, despite a higher incidence of dysphagia in the postoperative period linked to laparoscopic treatment and with an incidence of persistent dysphagia (1 year) coinciding between the conventional and laparoscopic techniques. This same study, when subjecting patients with postoperative dysphagia to HREM, found that most of them had Ineffective Esophageal Motility (IEM). Although the study’s methodology does not allow the establishement of cause-effect, it does draw attention to the relationship between IEM and postoperative dysphagia. The cause of post-operative dysphagia is still uncertain, with the possibility of it being related to motor disorders of the esophageal body or to the design of the fundoplication. In both situations, HREM seems to be the test of choice for assessing these patients’ eligibility for surgical treatment of GERD. However, as it is a test that uses recent technology and is still development, there is a certain heterogeneity in the devices and its use to assess patients who are candidates for surgery treatment of GERD. In parallel with the constant evolution of the technology in HREM devices, the systematization of the acquisition and interpretation of exams, currently carried out using the Chicago Classification 4.0 (CC4.0), has been constantly improved. One of the concerns of the Chicago Classification is precisely to identify patients at risk of post-fundoplication dysphagia and its parameters have been gradually trying to improve their efficiency in tracking these patients through the definition of IEM. One of the studies selected in this review attempts to evaluate the correlation between post-hiatoplasty and fundoplication dysphagia and IEM using the parameters established by the Chicago Classification 3.0 (CC3.0) and compare this same correlation using the definitions of IEM determined by CC4.0, its most recent version^9^. To this aim, a retrospective review was carried out of 206 patients who underwent HREM before and after laparoscopic Nissen fundoplication surgery. Dysphagia was identified in 18.4% of these patients and they were classified as having IEM using the CC3.0 and CC4.0 criteria. Using CC3.0 criteria, IEM was diagnosed in 41.3% of patients and in 35.4% of patients using CC4.0. The study concludes that the diagnosis of IEM was a reliable marker of post-fundoplication dysphagia, but the use of a more restricted diagnosis of IEM by CC4.0 criteria did not impact on the assertiveness of predicting post-fundoplication dysphagia compared to CC3.0, with both having similar sensitivity, specificity, accuracy and area under the ROC curve.

Historically, the first two studies attempting to evaluate HREM parameters as predictors of dysphagia after laparoscopic hiatoplasty and fundoplication were published in 2003 and 2005. Winslow et al. evaluated 168 patients in a clinical trial with the aim of characterizing the results after surgery for GERD in patients with “non-specific spastic disorders” of the esophageal body compared to patients who did not have it^10^. The authors raised the possibility that patients with esophageal motor disorders had more persistent esophageal symptoms after surgery for GERD. Thirty-six patients with motor disorders and 88 in the control group were assessed, and dysphagia was identified in 22 patients of all. A trend towards more frequent post-operative dysphagia was observed in patients with non-specific motor disorders (group with non-specific motor disorder 28% x 14% control group, *P=*0.061). However, this study was carried out using HREM parameters that are not currently used, and its factors have no applicability in the present reality. Even so, there was documentation of the relationship between non-specific esophageal motor disorders and dysphagia after laparoscopic hiatoplasty and fundoplication.

In 2005, Scheffer et al. evaluated, using HREM, what a successful hiatoplasty and fundoplication would look like in terms of food transit time through the esophagogastric junction (EGJ), the mechanisms for opening the EGJ and the relationship between these phenomena and the onset of dysphagia in the post-operative period^11^. Twelve patients with GERD who underwent laparoscopic hiatoplasty and total fundoplication were evaluated. The authors concluded that in this study there was no correlation between direct manometric parameters and postoperative dysphagia. It was found that cases of dysphagia were associated with prolonged transit time through the EGJ. However, it can be inferred that this is either the result of an esophageal body without sufficient motility to overcome the obstacle imposed by the fundoplication, or the result of a tight fundoplication, even with the motility of the esophageal body preserved, it is not possible to pass through the high-pressure zone resulting from the fundoplication.

A historical series carried out by Candice et al. was also ineffective in identifying manometric factors that predict dysphagia using HREM^12^. Analyzing the preoperative HREM of 14 patients with dysphagia after laparoscopic hiatoplasty and total fundoplication, it was found that nine of them already had dysphagia preoperatively and only five of them developed it after surgery. Considering preoperative clinical and manometric parameters, only the following were positively correlated with postoperative dysphagia: female gender (*P*=0.02) and preoperative dysphagia (*P*=0.05). In the same study, the authors concluded that post-operative alterations in the functional anatomy of the hiatus is the factor responsible for dysphagia after hiatoplasty and fundoplication. This study has some limitations, the main one being the small number of participants, although the statistical calculations show some statistical significance.

The absence of predictors of post-operative dysphagia in laparoscopic hiatoplasty and fundoplication was also observed in the retrospective study published by Siegal et al. in 2018^13^. They evaluated patients with GERD who underwent laparoscopic hiatoplasty and total fundoplication. These patients were divided into 4 groups: (1) patients who never had dysphagia; (2) patients who had only postoperative dysphagia; (3) patients who had preoperative dysphagia and persisted with it after surgery; (4) patients who had dysphagia resolved with surgery for GERD; totaling 94 patients. In conclusion, in patients without dysphagia before surgery and with acceptable esophageal body function, the HREM criteria could not predict the development of dysphagia in the postoperative period. Preoperative dysphagia was considered by the authors to be a common finding and it typically resolves after fundoplication in most cases, especially those with high manometric pressures. In cases of preoperative dysphagia in patients with low manometric criteria, dysphagia is more likely to persist and they may benefit from partial fundoplication, although more tests are needed to support this inference according to the authors. A limitation of this study is the fact that they divided the patients into 4 groups so that the number of individuals in each group was small. In addition, patients with relevant alterations in the HREM (Distal esophageal contraction amplitude <25 mmHg and percentage of esophageal body peristaltic waves <50%) were not included because the routine in this service is to perform partial fundoplication in these cases; it was one of the exclusion criteria. However, when evaluating the arm of the patients with dysphagia resolved by hiatoplasty and fundoplication, the authors noted that most of them had Distal Contractile Integral (DCI) measurements greater than 1000mmHg.s.cm (*P*=0.001), the latter being an important HREM factor for selecting patients with dysphagia to undergo total fundoplication. This is the first study to provide information from HREM that can be used as a criterion for selecting patients candidates to this surgical technique for the treatment of GERD. The authors state that DCI >1000 mmHg.s.cm is a factor related to good postoperative outcomes in terms of dysphagia resolution.

After a few studies showing little evidence of the applicability of the classic HREM parameters as predictors of dysphagia after hiatoplasty and fundoplication, arose the hypothesis of applying provocative tests during acquiring HREM to assess the capacity of esophageal motility to overcome the obstacle to esophageal emptying resulting from fundoplication. In 2013, Shaker et al. proposed the multiple rapid swallow test (MRST) to assess the contractile reserve of the esophagus^14^. They hypothesized that a suboptimal contraction in response to the MRST indicates a weak contractile reserve, which may be associated with chronic motility symptoms following antireflux surgery. To test this hypothesis, they first characterized the response to MRST in the entire esophageal body and, individually, in each segment of the esophageal musculature in healthy control patients. Using this criterion, the authors determined whether the MRST in the preoperative HREM in patients undergoing laparoscopic hiatoplasty and total fundoplication was able to identify patients with persistent dysphagia after surgery. Sixty-three patients were prospectively evaluated, all of whom had satisfactory manometric parameters to support total fundoplication. Early dysphagia was identified in 34 patients and late dysphagia (3 months postoperatively) in 18 of them. Besides reiterating the preoperative dysphagia as a predictor of postoperative dysphagia (*P*=0.0014), the authors conclude that the absence of increase in smooth muscle contraction (DCI of MRST/average DCI <1) after MRST is significantly more prevalent in patients who develop late postoperative dysphagia. This was the first study to recommend provocative testing as a predictor of postoperative dysphagia. The limitations of this study include a relatively small sample size, factors related to the patient and the technique used to perform the MRST, and the fact that some patients may not be able to perform the test satisfactorily.

The preoperative MRST of patients with GERD was also evaluated by Hazak et al. in 2019^15^. They conducted a prospective review of 157 patients, 136 of whom underwent laparoscopic hiatoplasty and total fundoplication with preoperative HREM. Postoperative dysphagia was identified in 101 patients, of whom 86 had early dysphagia (<6 weeks), 32 had preoperative dysphagia and 38 progressed to late dysphagia. Analysis of the data using the Kaplan-Meyer method shows that preoperative dysphagia, the appearance of early dysphagia in the postoperative period and the recurrence of HH are associated with clinically significant late dysphagia after surgery. However, only MRST with abscense of increase in esophageal body smooth muscle contraction was able to independently predict postoperative dysphagia (*P*=0.04). Limitations of this study include the fact that the authors did not routinely perform HREM on all patients in the postoperative period, but only on symptomatic patients; the frequency of PPI us by patients in the postoperative period was also not studied.

The most recent study published on the manometric predictors of dysphagia after hiatoplasty and fundoplication was carried out by Salvador et al. in 2024^16^. In this study, the aim was to establish the standards in HREM for evaluating an effective fundoplication and to determine how HREM can discern between a successful or unsuccessful fundoplication. A prospective analysis was carried out between 2010 and 2022 involving 132 laparoscopically operated patients who underwent total or partial fundoplication, depending on preoperative HREM factors. Five patients (all of whom underwent total fundoplication) had persistent postoperative dysphagia and the only preoperative manometric measurement that showed an association with the failure of anti-reflux surgery (symptoms and/or changes in pHmetry) was the percentage of normal contractions (*P*=0.011) and ineffective swallowing (*P*<0.001). No independent association emerged between preoperative manometric measurements and laparoscopic hiatoplasty and fundoplication failure in multivariate analysis.

## CONCLUSION

In light of the exposed, the data in the literature on manometric predictors of dysphagia after hiatoplasty and fundoplication are derived from somewhat heterogeneous studies, whether due to the number of probe channels used, the technology used to measure esophageal pressure (solid-state or water perfusion), the definitions of early, late or persistent dysphagia or even the methodology used to acquire and interpret HREM data. These facts make it impossible to carry out a meta-analysis, so a systematic review with a descriptive comparison of the various studies available in the medical literature is necessary.

With the advent of HREM and its greater detail of the EGJ, expectations for a better understanding of dysphagia after laparoscopic hiatoplasty and fundoplication have prompted studies to evaluate the role of this test in selecting patients who are candidates for surgical treatment of GERD, avoiding dysphagia in the postoperative period if possible. The first studies with this purpose failed to identify preoperative predictors of dysphagia. It was only in 2013 that Shaker et al. using MRST to assess the contractile reserve of the esophageal body, were able to demonstrate a manometric factor that predicted dysphagia in the postoperative period. Hazak et al. later also identified that patients with IEM who have little contractile reserve as demonstrated by MRST have a higher incidence of dysphagia in the postoperative period. In parallel, Siegal et al. in 2018 proposed, after evaluating patients with preserved esophageal motility, that patients with dysphagia present preoperatively, the finding of DCI>1000mmHg.s.cm is a predictor of dysphagia resolution after surgery. As a result, we now know that HREM not only helps to exclude other differential diagnoses of GERD, such as idiopathic achalasia, but is also a useful tool in defining the surgical strategy to be used in GERD patients who have IEM or dysphagia preoperatively.
